# Assessing the nutrition knowledge, beliefs, and behaviors of food pantry managers: implications for healthier food environments

**DOI:** 10.3389/fpubh.2025.1544413

**Published:** 2025-02-17

**Authors:** Sofia O. Sanchez, Katie Funderburk, Erin Reznicek, Robert R. Bubb, Andrew D. Frugé, Adrienne Duke-Marks, J. Benjamin Hinnant, Sondra M. Parmer

**Affiliations:** ^1^Alabama Cooperative Extension System at Auburn University, Auburn, AL, United States; ^2^College of Human Sciences, Human Development & Family Science, Auburn University, Auburn, AL, United States; ^3^College of Nursing, Auburn University, Auburn, AL, United States

**Keywords:** food pantries, nutrition environment, social determinants, community nutrition, SNAP

## Abstract

**Introduction:**

Food pantry managers play a key role in determining the food environment of the pantry, which can influence their clients’ dietary composition. However, their impact on pantry food environments remains understudied. We sought to understand food pantry managers’ nutrition knowledge, beliefs, and behaviors (KBBs) in this study.

**Methods:**

We surveyed 47 Alabama food pantry managers’ nutrition KBBs from November 2022 to November 2023. Validated and previously published tools used include the Consumer Nutrition Knowledge Scale, a diet beliefs scale, and a dietary-related consumer behavior questionnaire.

**Results:**

The majority of managers were college-educated (54%), white (74%), and female (74%), with a mean age of 60 ± 13 years and an average of 7 ± 5.5 years of experience in managing food pantries. Managers reported positive nutrition beliefs and behaviors but scored lower on objective nutrition knowledge. Exploratory analyses indicated moderate to very strong associations between KBBs, pantry characteristics, and neighborhood characteristics.

**Conclusion:**

Nutrition education for pantry managers, along with improved pantry infrastructural support, could play a key role in improving the healthfulness of the food provided.

## 1 Introduction

Food insecurity is a state of inadequate access to food to sustain active and healthy living and affects 13.5% of households in the United States (1). Food pantries exist to serve individuals in need of food resources and may provide wraparound (i.e., non-food) services, for example, literacy classes or housing assistance (2). Despite efforts made by pantries to provide food assistance, a number of studies document barriers to distributing healthy foods to clients, such as inadequate infrastructure, storage facilities, and reliance on donations (3–7).

Studies have explored the role of the food pantry manager and their perceptions of what constitutes an adequate pantry, focusing on the distribution methods and food composition (8–10). However, the number of available studies on this topic remains limited. In other food provision settings, such as retail environments, managers of food stores in low resource neighborhoods have reported mixed knowledge regarding the nutritional benefits of the foods they stock (11). As food pantry managers are in a position to procure, promote, and provide healthy foods to clients, it is important to understand their knowledge, beliefs, and behaviors (KBBs) around healthy eating and general nutrition.

Improving or altering food environments has been studied as a method to alter individual and community health behaviors (12). Food pantries are ideal settings to target dietary behaviors, as 16–66% of households that utilize pantries consist of at least one member with a diet-related chronic illness (13, 14). Additionally, the role of neighborhood characteristics has been studied in influencing food environments. For example, county-level characteristics, such as the percentage of the population with a high school education, poverty, and rurality, are negatively associated with the number of pantries in a given county (15). Another study documents a county’s resident demographics, such as the proportion of food insecurity, race ethnicity, and political affiliation as significant predictors of the number of pantries in a county (16). However, there are not yet any studies on neighborhood characteristics’ influence on the food environment of a pantry. Therefore, we sought to characterize the nutrition KBBs of food pantry managers in Alabama. A secondary, exploratory objective of this study was to determine whether a food pantry environment intervention and neighborhood characteristics of the pantry were associated with manager KBBs.

## 2 Methods

### 2.1 Study design

This cross-sectional study was part of a larger project, the Live Well Alabama Healthy Food Pantry Program (LWA HFP), which was a single-arm, open-label intervention evaluating the outcomes of food pantry nutrition environments in Alabama (*n* = 55). Following the completion of the food pantry intervention, managers of participating pantries were recruited from May 2023 to July 2023 to participate in a manager survey. Data were sent to a researcher for data entry, which was entered into an online, password-protected database. This study was approved by the Auburn University Institutional Review Board (protocol #22-128 EX 2303).

### 2.2 Measures

#### 2.2.1 Nutrition knowledge, beliefs, and behaviors

Manager KBB questions were adapted from a range of previously published data or validated instruments and were as follows: Nutrition knowledge was assessed through a 20-item validated instrument, the Consumer Nutrition Knowledge Scale (CoNKS) (17). The CoNKS was a composite assessment of nutrition knowledge that measures declarative nutrition knowledge, such as the awareness of a fact, and procedural nutrition knowledge, such as the awareness of a process. Each item on the instrument was a true or false question, e.g., “Oily fish (salmon, mackerel) contain healthier fats than red meat.” The instrument’s internal reliability was assessed using Cronbach’s alpha, where *α* = 0.73, demonstrating good internal reliability.

Nutrition beliefs were measured through nine questions adapted from the study previously published by Barratt et al. (18). An example of a question posed to pantry managers was, “Eating healthy food is expensive,” to which managers could select Likert scale-like responses, ranging from 1 to 4, with negative beliefs ranked 1 and positive beliefs ranked 4. Responses were summed to create a composite score.

Nutrition behaviors were measured by eight questions asking managers about dietary-related consumer behaviors. Managers marked the frequency, from “Always” to “Never seen” on a 7-point Likert-scale, the frequency with which they look for specific nutrients on food labels. The questions were from the 2017 to March 2020 version of the National Health and Nutrition Examination Survey (NHANES) (19).

Manager demographics were also captured through this survey, such as age, gender, marital status, years of experience as a manager, race ethnicity, and use of a federal nutrition assistance program within the previous 12 months to determine Supplemental Nutrition Assistance Program (SNAP) eligibility. These questions also align with demographic questions from the NHANES.

#### 2.2.2 Food pantry characteristics

Questions about the characteristics of the pantry were collected through the manager survey and were based on the study previously published by Yan et al. (8). These included the number of pounds of food distributed in the previous month, the total number of salaried employees, the total number of volunteers, the total number of freezers, and the total number of coolers.

#### 2.2.3 Neighborhood characteristics

To characterize a pantry’s local neighborhood characteristics, variables from the American Community Survey 5-year estimates of the 2020 U.S. Census, Area Deprivation Index (ADI), and Rural–Urban Commuting Area (RUCA) codes were selected (13, 20, 21). The ADI is a composite measure of neighborhood socioeconomic disadvantage and considers measures of income, education, employment, and housing quality. Scores range from 1 to 100, where 1 indicates the lowest disadvantage and 100 indicates the highest disadvantage (22, 23). The eight-digit zip codes of each food pantry were geocoded by matching with variables within the pantry’s zip code tabulation area (ZCTA) and used to match the corresponding tract-level ADI value. The ADI reliability coefficient is *α* = 0.95, demonstrating high internal consistency (25). Rural–Urban Commuting Area (RUCA) codes are based on U.S. census tracts that measure population density, urbanization, and daily commuting (24). Using the most recent RUCA codes, pantries were classified as urban or rural using methods as described by Caspi et al. (13) where 1–3 = urban, 4–10 = rural.

#### 2.2.4 Statistical analysis

Statistical analyses were conducted using SPSS version 29 (Armonk, NY: IBM Corp) and jamovi version 2.5 (Sydney, Australia: The jamovi project). Descriptive statistics were obtained for participant characteristics and survey responses. Correlations were conducted using Pearson’s *r* for normally distributed data and Kendall’s Tau B for non-normally distributed data. Due to the small sample size, correlations were evaluated for measures of effect. An exploratory factor analysis (EFA) was conducted on the 9-item scale that measured nutrition beliefs among 37 participants with complete responses. An oblique rotation was utilized to increase external validity and aid in interpretation. Alpha was set at 0.05 for all analyses.

## 3 Results

**Figure 1:**
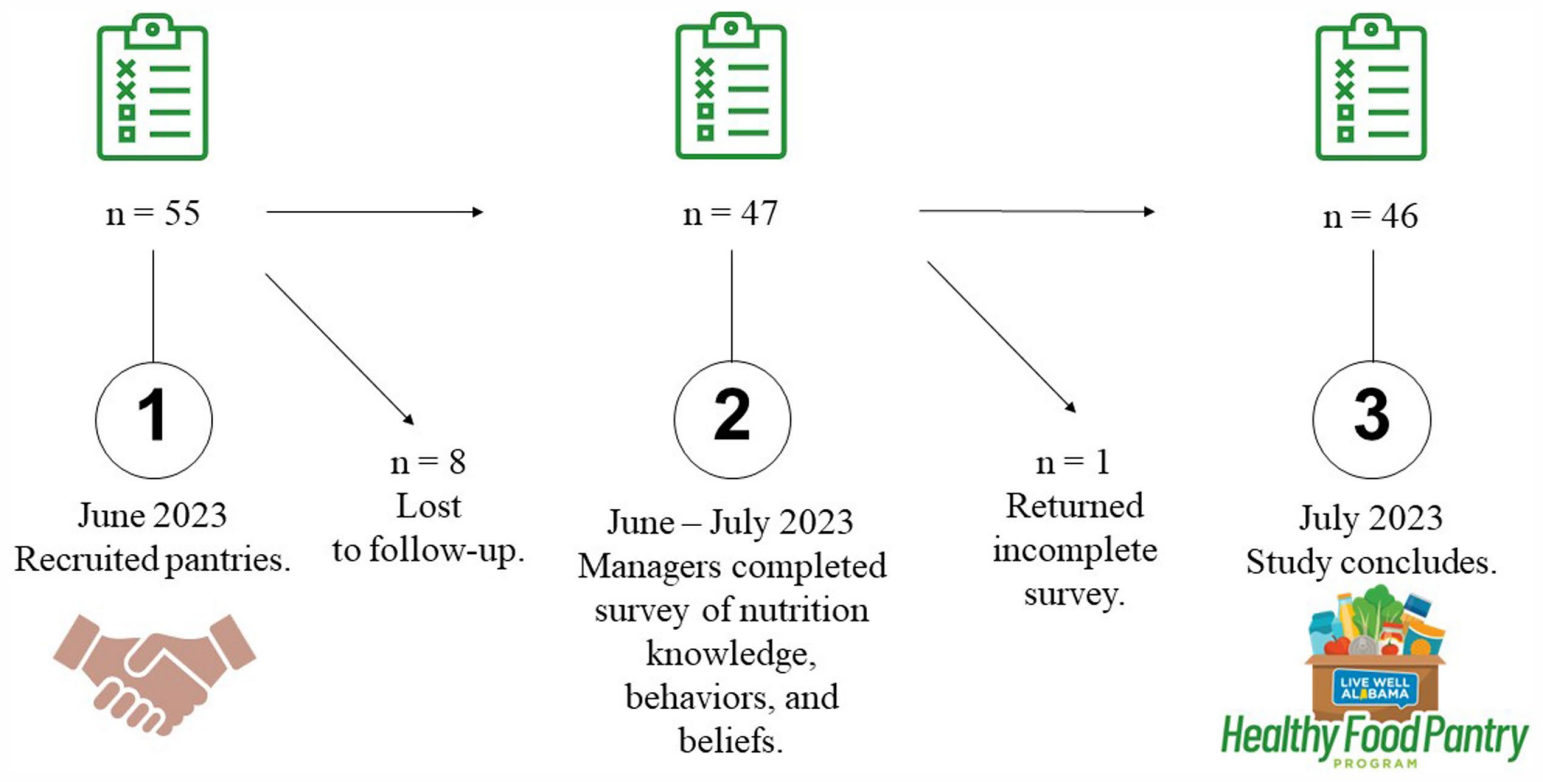
Schematic of live well Alabama healthy food pantry program participant.

Fifty-five participants were recruited for the study. Of these, 47 consented, and 46 provided complete responses to the questionnaires (see Figure 1). Managers were predominantly college-educated (54%), white (74%), and female (74%), with a mean age of 60 ± 13 years and an average of 7 ± 5.5 years of experience in managing food pantries. Complete participant demographics are presented in Table 1. Descriptive characteristics of the pantries can be found in Supplementary Table 1. Over 80% of participants managed pantries supported by faith-based institutions.

**Table 1:** Participant demographics.

*n* = 47	
Gender (woman; man)	35; 12
Age in years (M ± SD)	60 ± 13
SNAP eligible	7 (15)
Education	
High school or equivalent	7 (15)
Associate’s degree	8 (17)
Some college, no degree	6 (13)
Bachelor’s degree or higher	25 (54)
Unknown	1(1)
Marital status	
Single, never married	5 (10)
Divorced or separated	6 (13)
Widowed	7 (15)
Married or living with a partner	28 (60)
Unknown	1(1)
Years of experience as a manager	7 ± 5.5
Race	
Black	12 (26)
White	35 (74)

Data are presented as mean ± standard deviation (M ± SD) and total count (percent of sample).

### 3.1 Manager KBBs

A list of each nutrition knowledge question and the percentage of correct responses are presented in Supplementary Table 2. The average number of correct responses was 15 out of 20 questions, with incorrect answers related to fat type and dietary composition of foods.

Inspection of the scree plot and Lautenschlager values (26) of the nutrition beliefs EFA with orthogonal rotation revealed two components, with six items loading on component one (questions 1, 2, 6, 7, 8, and 9) and five items loading on component two (questions 3, 4, 5, 6, and 7). It was determined that component one measured personal health beliefs and component two measured beliefs of health expertise. The frequency of responses for each nutrition belief question and the percentage of responses are presented in Supplementary Table 3. The frequency of responses for each nutrition behavior question and the percentage of responses are shown in Table 2.

**Table 2:** Nutrition behaviors (*n* = 46).

	Always	Most of the time	I do not know	Sometimes	Rarely	Never	Never seen
Use expiration dates such as “use by” or “sell by.”	15 (33)	17 (37)	0	5 (11)	6 (13)	3 (7)	1 (2)
Use the Nutrition Facts panel.	15 (33)	16 (35)	0	11 (24)	2 (4)	2 (4)	1 (2)
Use the list of ingredients.	14 (30)	17 (37)	0	11 (24)	4 (9)	1 (2)	0
Use information on the serving size.	12 (26)	15 (33)	0	9 (20)	5 (11)	4 (9)	0
Use information on the number of servings in the package.	15 (33)	11 (24)	0	11 (24)	3 (7)	6 (13)	1 (2)
Use the calorie information.	15 (33)	13 (28)	0	13 (28)	3 (7)	3 (7)	0
Use information on sugars.	15 (33)	18 (39)	0	10 (22)	2 (4)	2 (4)	0
Use information on sodium.	14 (30)	17 (37)	0	6 (13)	5 (11)	5 (11)	0

Responses to the nutrition behaviors survey with percentages of respondents in parentheses. Managers were asked, “When deciding what to buy for yourself, how often do you…”.

The correlations between manager KBBs, pantry characteristics, and the local neighborhood of the pantries are presented in Table 3. Among the pantry characteristics, the number of volunteers demonstrated a moderate positive correlation with overall manager beliefs (*τ*_b_s = 0.203). The pounds of food distributed, the number of volunteers, and the number of coolers demonstrated a moderate correlation with expert health beliefs. The number of coolers was positively correlated with health expertise beliefs (*τ*_b_ > 0.2).

**Table 3:** Correlations between manager KBBs, pantry characteristics, and neighborhood characteristics.

	Beliefs	Knowledge	Behavior	Expert beliefs	Health beliefs
Pantry characteristics—manager survey					
Pounds of food distributed per month	0.118	0.050	0.065	**0.212**	0.047
Number of salaried employees	0.034	−0.041	−0.070	−0.025	0.160
Number of volunteers	**0.203**	0.149	0.145	**0.205**	0.123
Number of freezers	0.082	−0.127	0.070	0.178	−0.031
Number of coolers	0.141	0.088	−0.096	**0.244**	0.006
Neighborhood characteristics					
Area deprivation index	−0.184	**−0.309**	0.058	−0.146	−0.198
Urbanicity (RUCA)	0.055	0.050	−0.048	0.077	0.102
Female, %	0.141	0.097	0.117	0.107	0.189
Median age	0.069	0.103	−0.033	0.131	0.061
Under 18, %	−0.024	0.070	**0.225**	−0.051	−0.082
65 or older, %	0.091	0.069	0.053	0.154	0.110
White, %	0.175	0.191	0.034	0.150	0.156
Black, %	−0.145	**−0.206**	0.020	−0.106	−0.148
Multi-ethnicity, %	0.166	−0.138	0.008	**0.227**	0.139
Latino, %	0.117	−0.026	−0.016	0.058	0.015
Number of housing units	0.020	0.056	0.126	−0.051	0.054
Mean commute time, minutes	−0.031	−0.135	0.019	0.020	−0.035
No vehicle	**−0.243**	−0.040	−0.009	**−0.272**	−0.136
SNAP participation	−0.191	**−0.228**	−0.069	−0.119	**−0.232**
Median household income	**0.280**	0.114	0.118	**0.228**	**0.351**
Median housing cost	0.092	0.067	0.100	0.022	0.104
At least 1 computing device	**0.432**	0.060	−0.052	**0.372**	**0.434**
No Internet	**−0.247**	−0.132	0.011	−0.156	**−0.252**
Less than high school	−0.108	−0.081	−0.007	0.044	−0.16
Undergraduate	0.195	0.100	0.006	0.129	**0.257**
Graduate or higher	0.170	0.087	0.044	0.115	**0.202**

Moderate or greater effect sizes are bolded, where τ is approximately 0.26 for Kendall’s Tau B correlation (non-normally distributed data). Otherwise, Pearson’s r is used.

There were positive, moderate-to-very strong correlations between KBBs and neighborhood characteristics. When measured as one construct, beliefs were positively moderately correlated with median household income and having access to at least one computing device, but negatively correlated with having access to a vehicle and the Internet. When split into two constructs, health expertise beliefs were positively correlated with the percentage of residents identifying as multi-racial, median household income, and access to a computing device, but moderately negatively correlated with lack of vehicle ownership. Personal health beliefs were positively correlated with median household income, access to a computing device, and undergraduate degree, and negatively correlated with SNAP participation and Internet access. Nutrition knowledge was negatively correlated with ADI and the population receiving SNAP. Nutrition behaviors were positively correlated with the population under 18 years of age.

## 4 Discussion

The primary finding of this study was that managers reported positive beliefs and behaviors regarding nutrition but tended to score lower on objective nutrition knowledge. The second finding was that there were several moderate-to-very strong associations between pantry manager nutrition KBBs, pantry characteristics, and the local community surrounding a food pantry.

### 4.1 Manager KBBs

Managers generally held positive beliefs about nutrition. Out of 44 respondents, 41 (93%) reported that they “disagree” or “strongly disagree” with the statement “I get confused over what’s supposed to be healthy.” Out of 37 respondents, 35 (95%) reported that they “agree” or “strongly agree” with the statement, “I would feel confident if I was giving advice about healthy eating.” However, our study demonstrates that managers reported positive nutrition KBBs while scoring low on objective measures of the pantry’s nutrition environment, and vice versa. Our study supports the literature by demonstrating that factors such as pantry infrastructure, budgets, or resources available that enable healthy provisions influence pantry practices (10, 27). These factors may serve as stronger determinants of a pantry’s nutrition environment than the managers’ nutrition KBBs.

Our study also showed that a manager’s perceived beliefs about nutrition may not match objective nutrition knowledge. Nutrition knowledge scores were as low as 8 out of 20 (40% correct rate), with a mean total score of 15.2 (76%) and a mode of 15 (75%), indicating an opportunity to increase nutrition knowledge among the food pantry manager population in Alabama. Importantly, the majority (86%) of participants, though not all, agreed or strongly agreed with the statement “Providing healthy foods is part of my job,” demonstrating that the majority of managers feel some form of responsibility to provide healthy foods. This further demonstrated an opportunity to engage and collaborate with managers to support or implement policy, systems, and environmental changes to improve the pantry nutrition environment. The nutrition facts label provides information about the nutrition content of certain foods and can help guide food decisions. Pantry managers reported similar use of the nutrition facts label compared to the general public. In the NHANES survey data, 79% of adults report regularly using the label when making food purchasing decisions compared to 88% in our sample (28).

Based on effect sizes, there were several associations between manager nutrition KBBs and pantry characteristics. Specifically, nutrition beliefs were positively associated with the number of volunteers. This may suggest that managers who value nutrition prioritize healthy practices in the pantry, which translates to seeking or accepting a greater number of volunteers to support these practices. Indeed, validated instruments that measure pantry food environments incorporate aspects of volunteer training and customer service effectiveness—key pantry operations that are led by volunteers and overseen by managers (29). While not captured in this study, this may also be influenced by pantry size, where larger pantries with greater demands are supported by a larger number of volunteers.

Additionally, nutrition expertise beliefs were associated with pounds of food distributed per month, the number of volunteers, and the number of coolers in a pantry. Other studies describe having adequate refrigeration as a reported barrier to distributing healthy foods by food pantry personnel (27, 30). Thus, managers working to overcome this barrier by making refrigeration available may have an increased level of expertise, such as applying for grant funding or identifying supportive community partners. This also demonstrates the importance of financial support for pantries, as pantries with greater funding are better able to purchase infrastructure for foods that need cold storage.

These findings slightly diverged from another sample of pantries in the southeastern United States where pantries reported having adequate freezer storage more than adequate refrigerator storage (31). However, pantry cold storage may not align with clients’ ability to receive perishable foods. In a descriptive study of a sample of food pantry clients, 87% had a refrigerator, whereas 61% had a freezer (32). This suggests that managers with greater levels of expertise may be selecting foods that reflect increased skills in understanding the needs of their clients.

The demographic profile of the average manager was a married, white woman, approximately 60 years old, with a bachelor’s degree or higher level of educational attainment. Of note, Alabama is among the top 10 U.S. states with the highest Black or African American population (29.8%), with some counties being home to up to 82% Black or African American residents (33). Few studies capture manager demographics; however, our sample’s demographics align with a sample of Oklahoma pantry personnel where it was found that the majority of pantry personnel were, on average, 61 years old, female, and white (34). These demographic differences between managers and clients may suggest differences in food preferences or differences in the cultural relevance of different foods. This finding aligns with other previous studies demonstrating mismatches between what clients desire and what managers believe they should receive, including in pantries in the Southern United States (35). Future studies of food pantries could consider measuring managers’ perceptions of the adequacy of the foods they serve.

### 4.2 Strengths and limitations

This study is not without limitations. First, a number of correlations demonstrated a weak-to-moderate (*r* ≥ 0.1–0.04, *τ*_b_ ≥ 0.6–0.26) effect. However, this was exploratory and underpowered to determine significant regression findings. With 21 participants providing complete data. With a larger sample size, there may have been additional significant findings. Finally, our cross-sectional observation limited the ability to determine changes that resulted from the food pantry environment intervention.

Increasing the quantity of fruits and vegetables in food assistance settings has been studied using various implementation strategies. Some studies (36–38), though not all (39), find improvements in client self-reported diet quality following food pantry environment interventions. Future studies of food pantry nutrition environments or food pantry managers could benefit from conducting pre- and post-intervention assessments to draw inferences about changes in manager KBBs alongside pantry measures.

### 4.3 Application for practice

This study sought to measure the nutrition KBBs of food pantry managers’. Our findings suggest that offering or conducting nutrition education can support and increase the nutrition knowledge of pantry personnel. The Supplemental Nutrition Assistance Program – Education largely operates through cooperative extension services and offers nutrition education along with multi-level community interventions to improve nutrition (40). Given that extension offices are in or close to nearly every county (41), forming pantry-SNAP-Ed partnerships could be particularly beneficial for rural and under-resourced areas, in addition to the clients they serve.

## Data Availability

The datasets presented in this study can be found in online repositories. The names of the repository/repositories and accession number(s) can be found at: https://figshare.com/s/75d5a5b87dd58f5c5163.
