# Supplementary material for: Assessing the nutrition knowledge, beliefs, and behaviors of food pantry managers: implications for healthier food environments
Source: Front Public Health. 2025 Feb 17;13:1544413. doi: 10.3389/fpubh.2025.1544413 (PMC11873097; doi:10.3389/fpubh.2025.1544413)
Supplement: Supplementary file 1 [file Data_Sheet_1.docx]

Supplementary Material

# Supplementary Data

|  |  |  |  |
| --- | --- | --- | --- |
|  | **Supplementary Table 1. Pantry Characteristics** |  |  |
|  | n = 47 |  |  |
|  | **Pantry type** | **n (%)** |  |
|  | School | 0 (0) |  |
|  | Faith-based institution | 38 (80) |  |
|  | Community organization | 9 (19) |  |
|  | **Descriptive characteristics** | ***M ±* SD** |  |
|  | Pounds of food distributed | 26418 ± 112717 |  |
|  | Salaried employees | 1 ± 2 |  |
|  | Volunteers | 25 ± 42 |  |
|  | Freezers | 4 ± 3 |  |
|  | Coolers | 2 ± 2 |  |
|  | Note: Descriptive data are presented as mean ± standard deviation. | |  |
|  |  |  |  |
|  |  |  |  |

|  | **Supplementary Table 2. Consumer Oriented Nutrition Knowledge Questionnaire** |  |  |  |
| --- | --- | --- | --- | --- |
|  |  | True/false | % Correct responses |  |
|  |  |  |  |  |
|  | A salad dressing made with mayonnaise is as healthy as the same dressing made with mustard. | F | 98 |  |
|  | Lentils contain only few useful nutrients, therefore their health benefit is not great. | F | 96 |  |
|  | A balanced diet implies eating all foods in the same amounts. | F | 91 |  |
|  | A scoop of chocolate ice cream is just as healthy as a scoop of lemon sorbet. | F | 91 |  |
|  | Oily fish (salmon, mackerel) contain healthier fats than red meat. | T | 91 |  |
|  | The same amount of steak and chicken breast contain an equal amount of calories. | F | 89 |  |
|  | Fat contains fewer calories than the same amount of fiber. | F | 87 |  |
|  | If cream is whipped it contains less calories than in its liquid form. | F | 87 |  |
|  | For a healthy diet, dairy products should be consumed in the same amounts as fruit and vegetables. | F | 87 |  |
|  | The same amount of sugar and fat contain an equal amount of calories. | F | 87 |  |
|  | If you have eaten high-fat foods, you can reverse the effects by eating apples. | F | 83 |  |
|  | A healthy meal should consist of half meat, a quarter vegetables and a quarter side dishes. | F | 76 |  |
|  | The health benefit of fruit and vegetables lies alone in the supply of vitamins and minerals. | F | 74 |  |
|  | Fat is always bad for your health; you should therefore avoid it as much as possible. | F | 72 |  |
|  | A sandwich with mozzarella contains as many calories as the same sandwich with Swiss cheese. | F | 67 |  |
|  | Skimmed milk contains fewer minerals than full-fat milk. | F | 65 |  |
|  | Pasta with tomato sauce is healthier than pasta with mushroom and cream sauce. | T | 59 |  |
|  | Brown sugar is much healthier than white sugar. | F | 48 |  |
|  | Bacon contains more calories than ham. | T | 43 |  |
|  | To eat healthily, you should eat less fat. | F | 30 |  |
|  |  |  |  |  |
|  | Note: The 20 items of the nutrition knowledge scale, their correct answer, and the percentage of respondents answering correctly (n= 46). Data are sorted in descending order of correct response rates. T: true, F: false. | | |  |
|  |  |  |  |  |
|  |  |  |  |  |

|  | **Supplementary Table 3. Nutrition Beliefs (n = 46)** |  |  |  |  |  |  |  |  |  |  |  |
| --- | --- | --- | --- | --- | --- | --- | --- | --- | --- | --- | --- | --- |
|  |  | Strongly disagree | % strongly disagree | Disagree | % disagree | Undecided | % undecided | Agree | % agree | Strongly agree | % strongly agree |  |
|  | 1. I get confused over what's supposed to be healthy and what isn't. | 26 | 100 | 15 | 42 | 3 | 7 | 2 | 4 | 1 | 2 |  |
|  | 2. Eating healthy food is expensive. | 1 | 4 | 8 | 22 | 4 | 9 | 26 | 57 | 8 | 17 |  |
|  | 3. Healthy foods are enjoyable. | 1 | 4 | 4 | 11 | 4 | 9 | 23 | 50 | 15 | 33 |  |
|  | 4. The tastiest foods are the ones that are bad for you. | 8 | 31 | 19 | 53 | 11 | 24 | 7 | 15 | 2 | 4 |  |
|  | 5. Healthy eating is just a trend. | 20 | 77 | 24 | 67 | 3 | 7 | 0 | 0 | 0 | 0 |  |
|  | 6. As long as you do enough exercise you can eat what you like. | 17 | 65 | 19 | 53 | 7 | 15 | 4 | 9 | 0 | 0 |  |
|  | 7. Experts never agree about what foods are good for you. | 7 | 27 | 19 | 53 | 15 | 33 | 4 | 9 | 2 | 4 |  |
|  | 8. Providing healthy foods is part of my job. | 1 | 4 | 3 | 8 | 4 | 9 | 22 | 48 | 17 | 37 |  |
|  | 9. I would feel confident if I was giving advice about healthy eating. | 0 | 0 | 2 | 6 | 10 | 22 | 27 | 59 | 8 | 17 |  |
|  |  |  |  |  |  |  |  |  |  |  |  |  |
|  | Note: The nutrition beliefs survey responses with percentage of respondents. The allocation of the nutrition beliefs constructs are as follows: questions 1, 2, 6, 7, 8, and 9 correspond to health expertise. Questions 3, 4, 5, 6, and 7 correspond to health perceptions. | | | | | | | | | |  |  |
|  |  |  |  |  |  |  |  |  |  |  |  |  |
